# Commercial and on-farm microbial bioinputs production systems: challenges, opportunities and current Brazilian regulatory framework

**DOI:** 10.1007/s42770-026-01955-3

**Published:** 2026-05-11

**Authors:** Vanessa Lucas Xavier, Helson Mario Martins do Vale, João Ricardo Moreira de Almeida

**Affiliations:** 1 Graduate Program of Microbial Biology, Institute of Biology, University of Brasília, Brasília, Brazil; 2 Department of Phytopathology. Institute of Biology, University of Brasília, Brasília, Brazil; 3 Microbial Genetics and Biotechnology Laboratory, Embrapa Agroenergy, Brasília, Brazil

**Keywords:** Bacterial bioinputs, Fungal bioinputs, Biofactories, Legislation, Biological security, Food security

## Abstract

Microbial bioinputs have emerged as an innovative solution to more sustainable agriculture, with microorganisms playing diverse roles in plants. The increasing adoption of bioinput requires efficient production systems, which can be industrial facilities or on-farm biofactories - self-use units installed on rural properties. Thus, evaluating the opportunities and potential risks associated with bioinput production, particularly those based on bacterial and fungal strains, is crucial. This work presents a review of the on-farm production of bioinputs in Brazil. First, the Brazilian regulatory framework for bioinputs and the environmental and economic advantages of using bioinputs are summarized. Then, opportunities and challenges of bioproduct on-farm production are reviewed and discussed. The results highlight that producer and environmental safety, as well as risks to public health due to microbiological contamination in biofactories, appear as the main challenges in on-farm production. Indeed, the propagation of non-target microorganisms, which can be potentially pathogenic to humans, such as *Escherichia coli* and *Salmonella* sp., has been reported in various studies. In addition, the success of bioinputs produced on-farm depends on the ability of the biofactories to properly propagate the target microorganisms and guarantee the quality of them. For this, certain parameters, such as viability, cell counting, identity, and other characteristics, must be analyzed during the manufacturing process. In conclusion, the growth trend of bioinput adoption and the risks associated with this type of product require adequate management, including the adoption of quality control in the production process that considers biological safety parameters.

## Introduction

The conventional model of agriculture, widely used in recent decades, employs excessive amounts of synthetic pesticides and chemical fertilizers. Due to its negative environmental and human health impacts, more sustainable practices have been developed to guarantee productivity while avoiding soil degradation, water body contamination, biodiversity loss, and greenhouse gas (GHG) emissions [1]. Microorganisms are essential in this context, promoting plant growth and protection against biotic and abiotic stress, acting as pest control, solubilizing nutrients and fixing nitrogen. They have been identified and used in agriculture as bioinputs, substituting or reducing the use of synthetic and chemical counterparts.

Bioinputs can be broadly defined as animal, plant, or microbial-based agricultural products, processes, and technologies employed in agriculture, livestock, forest, and aquatic systems to improve product production, storage, and processing [2]. Most of the bioinputs currently used in agriculture are based on living microorganisms, such as bacteria and fungi, but there are also inputs consisting of enzymes, plant extracts, plant hormones, macrobiological agents (mites, insects, and nematodes), and secondary metabolites [3]. The current scenario in the world’s agriculture and, more specifically, in Brazil, signals an increase in the adoption of bioinputs. It is estimated that the Brazilian market for bioinputs used in agriculture grew by 15% in the 2023/24 harvest, and sales of these inputs in the country totaled U$ 930 million, based on the final price for the farmer [4]. Regarding planted areas in Brazil, 25 million hectares have been treated with bioinputs [5].

With the significant advances in the development and successful application of bioinputs in agriculture, the demand for their production has grown dramatically in recent years. The bioinputs used in agriculture are mainly based on a few bacteria and fungi species, with an outstanding number of products based on *Bacillus* sp., *Metarhizium anisopliae*,* Beauveria bassiana* and *Trichoderma* sp. (Table 1) [6, 7]. In addition, other microbial species from different biodiversity sources have been identified and evaluated for future use [8, 9]. The relatively easy obtention and propagation of previously identified microbial strains further increase their use since they can be acquired as a commercial formulation or produced in biofactories – the so-called on-farm production. Indeed, the two systems for producing bioinputs are becoming increasingly established in Brazil. In commercial manufacturing, production takes place in the equipment and facilities of the manufacturing plants of national and multinational companies. On-farm production involves manufacturing for own use in units installed on rural properties [7, 10].

**Table 1:** Commonly employed species as bioinputs in agriculture and their function. (Source: MAPA [6])

Specie	Functions
*Bacillus subtilis*	Bactericide, nematicide, plant growth-promotion
*Bacillus thuringiensis*	Insecticide
*Bacillus amyloliquefaciens*	Bactericide, fungicide, nematicide
*Bacillus licheniformis* *Beauveria bassiana*	Nematicide Insecticide, acaricide
*Metarhizium anisopliae*	Insecticide, acaricide
*Trichoderma harzianum*	Fungicide
*Trichoderma asperellum*	Fungicide, nematicide
*Azospirillum brasilense*	Plant growth-promotion
*Bradyrhizobium japonicum*	Plant growth-promotion

While on-farm production can reduce production costs and make bioinputs more accessible, the functionality and purity of the target microorganism may not meet the same standards as those produced commercially. This approach often lacks the stringent quality and regulatory standards that are typically associated with commercial production [11]. In this context, this work summarizes the advantages and makes a counterpoint by detailing the potential risks involved in on-farm bioinput production. Additionally, it demonstrates the challenges and prospects for producing on-farm bioinputs, including the regulatory framework in Brazil. To this end, it briefly reviews the evolution of bioinputs, discusses the advances in their adoption, and how they can contribute to global food security. Finally, it discusses the advantages and potential risks involved in the on-farm production of bioinputs.

Based on the current Brazilian context, this work presents a hypothesis that on-farm production of microbial bioinputs represents a strategically promising pathway toward sustainable agriculture, but that its large-scale consolidation critically depends on three interdependent pillars: (i) effective regulatory oversight, (ii) implementation of quality control and biosafety practices within biofactories, and (iii) mechanistic understanding of microbial propagation, interaction and stability. Without these pillars, on-farm biofactories may become sources of biological, environmental and public health risks rather than drivers of sustainability. Therefore, this review evaluates the opportunities and risks of on-farm bioinput production in Brazil under this conceptual framework.

## Bioinputs usage: alignment with global initiatives for sustainable agriculture

The world faces the duality of producing food to feed a population expected to grow to 9.7 billion by 2050 while promoting ecological balance through sustainable practices. The agricultural model that has been practiced for decades, whose main objective is to maximize productivity through the massive use of pesticides and chemical fertilizers, has reached a level of unfeasibility [12, 13].

This conventional way of producing food threatens the economy, affects human health due to chemical residues in food and the toxic effects on farmers, and damages the environment by depleting natural resources, degrading the soil, inducing pest resistance, and emitting greenhouse gases. In short, synthetic inputs harm human, animal and environmental health [12, 13].

As a result, it has been necessary to rethink, change course, and invest in new strategies to combine high food production with sustainability. The advent of bioinputs and the awareness that this is a viable path has brought new perspectives for sustainable and efficient agribusiness. Nowadays, bioinputs have been considered essential biotechnological tools for guaranteeing food security worldwide [14].

The idea is not to completely replace chemical products with biological ones. Instead, a gradual complementation of the practices adopted with an increase in bio-based inputs is expected. Investments in research and development have resulted in innovations in this field, with large chemical companies gradually entering this niche market. More and more value-added biotech products have been launched, reinforcing the trend towards this approach; this is further evidence that this is the future of agriculture [10].

Specifically in Brazil, the bioinput market is developing rapidly, even faster than the global growth rates for this sector [1]. Estimates predict growth in the bioinputs segment by 16.6% per year in treated areas up to 2027/28 crop season in Brazil, corresponding to the highest growth rate within the agricultural inputs sector [15]. This Brazilian result represents approximately a quarter of the global bioinput growth, with an expansion speed of three to four times greater than the United States and Europe [15].

Another reason for using bioinputs is that they align with the Sustainable Development Goals (SDGs) of the United Nations. Policies to encourage the use of bioinputs and the technological transition practiced by companies can help achieve the goals of the 2030 Agenda, making sustainable agriculture possible. Specifically, bioinputs are related to and contribute to some SDGs such as “Zero Hunger and Sustainable Agriculture”, “Health and Well-being”, “Life in Water”, “Life on Land”, and “Industry, Innovation and Infrastructure” [2, 16].

In the Brazilian context, the importance of inoculants and biofertilizers within the scope of the National Fertilizer Plan 2022–2050 and their role in guaranteeing food security in Brazil and the world is highlighted. The Plan’s actions include encouraging innovation, increasing the supply of new products from emerging chains, and increasing the production and supply of organic and organo-mineral fertilizers [17]. With this, new emerging products and processes, such as organic and organo-mineral inputs and bioinputs, bioproducts, bioprocesses and biomolecules may help to reduce the dependence of Brazilian agribusiness on traditional inputs and imports from other countries, such as China and Russia [18]. Indeed, Brazil imports around 85% of fertilizers used in agriculture [17, 18]. Thus, the National Fertilizer Plan 2022–2050 is a state public policy aimed at reducing the country’s vulnerability and contributing to food security.

Another contribution of bioinputs is their convergence with the One Health principles established and widely disseminated by the World Health Organization (WHO). One Health foresees the need for harmony and interdependence between humans, animals, and the environment. The imbalance of one component has an impact on the others. It is well established that anthropogenic activities, such as the excessive use of chemical inputs on crops in recent years, have had a negative impact on the soil microbiota, resulting in a significant loss of beneficial relationships between microorganisms and plants [19]. Thus, bioinputs are considered to be a way of restoring environments, rescuing healthy soil conditions and stimulating the inherent capacity of these soil’s microbial community to reduce the incidence or severity of soil-borne plant diseases, with the consequence of more sustainable, robust and stable agricultural production to guarantee food security, with the supply of innocuous food in sufficient quantity for the entire population of the planet [19, 20].

###  Evolution of bioinputs

**Fig. 1:**
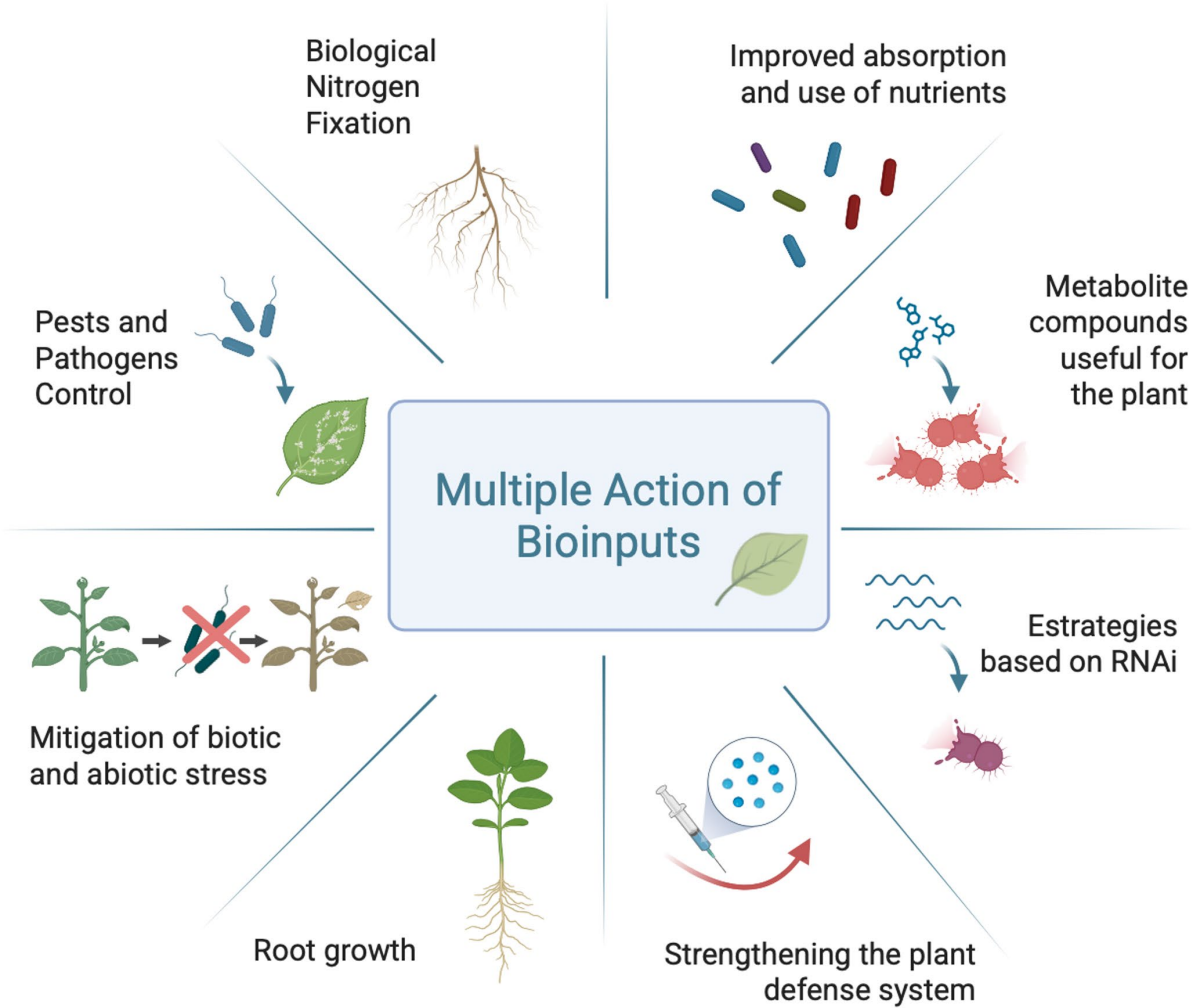
Multiple actions performed by bioinputs in agriculture

The concept of bioinputs is quite broad and is associated with various other terms, including “biobased product” and “bioproduct”. Furthermore, it can be said that there is no consensus in the literature regarding the definition and range of applications of these bioinputs [2]. They can perform a variety of functions in agriculture (Fig. 1), often simultaneously, such as strengthening the plant’s defense system, controlling pathogens and pests, mitigating biotic and abiotic stress, improving nutrient absorption, promoting biological nitrogen fixation and root growth, among other actions [21, 22].

Globally, bioinputs represent the latest technological strategies to promote sustainable agriculture by minimizing the use of fertilizers and synthetic pesticides [23]. In this context, there is evidence that bioinputs are on a growing trend. There has been a significant increase in the number of registrations of bio-based products in Brazil in recent years. By March 2026, the Ministry of Agriculture and Livestock (MAPA) had registered 834 products for phytosanitary purposes, or biocontrol, and 975 inoculants [24]. Phytosanitary purpose comprehends products intended for the prevention, suppression, eradication, or mitigation of pests, pathogens, and other organisms harmful to plants and plant products, while an inoculant is understood to be a product that contains microorganisms with a beneficial effect on plant development, that favors plant growth and performance [24].

**Fig. 2:**
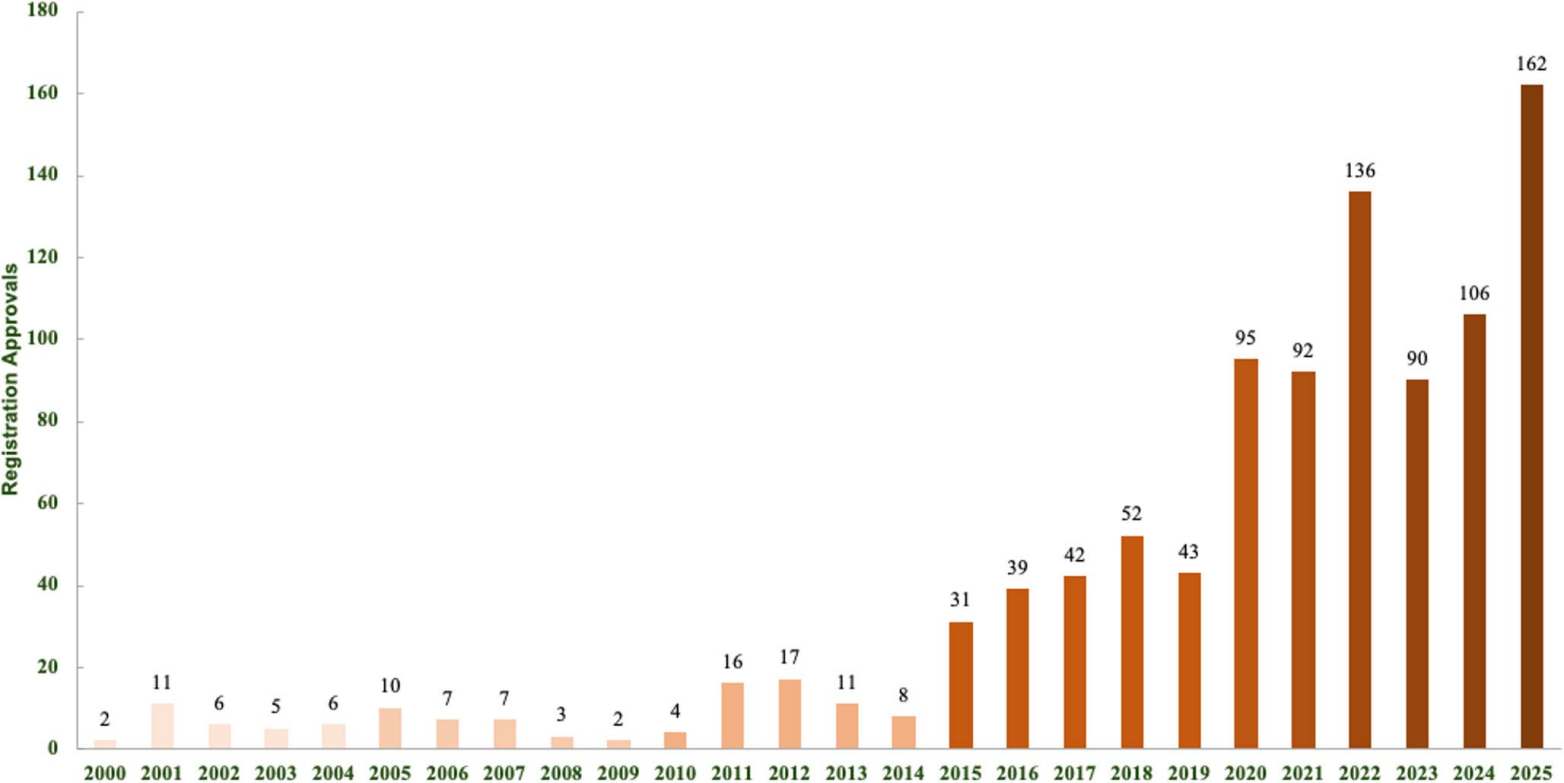
Evolution of the number of registered bioinputs for phytosanitary applications in the Brazilian Ministry of Agriculture and Livestock [6]

The data on phytosanitary products demonstrate a high increase in the number of registered products in recent years, with a historical record of 162 product approvals in 2025 (Fig. 2). The registered plant protection products include biological, microbiological, semiochemical, plant extracts, growth regulators, and products approved for use in organic farming [6]. It is noteworthy that half of the registrations occurred in the last five years, and most of them are based on living microorganisms (Table 1) [25].

The incorporation of bioinputs into the portfolios of major companies, the emergence of numerous start-ups, and the on-farm production of bioinputs for self-use [1, 26] demonstrate that sustainable practices are essential to prevent biodiversity loss and the degradation of Brazilian biomes, especially when compared with the excessive use of chemical inputs.

Additionally, one must consider the expansion of cultivated areas in Brazil where bioinputs are utilized, involving both small and large properties [27, 28]. Brazil is a global leader in adopting biocontrol techniques, with 55% of agricultural producers implementing them [5].

Research and adoption of bio-based products has been going on for decades. From a historical perspective, the use of a bacteria group called rhizobia capable of fixing nitrogen in soybean crops through symbiosis, led to a replacement of the use of N-fertilizers. Research began in the 40 s; effective strains were selected, farmers initiated the use of the inoculants, provided by the government of Rio Grande do Sul, and inoculants started to be produced in the 50 s by the first private Brazilian inoculant industry [29].

Farmers adopted this technology since 1960 as they realized the benefits associated with it, and Brazil became a benchmark and one of the largest producers of inoculants in the world [29]. It was estimated that replacing synthetic nitrogen fertilizer (urea) with biological nitrogen fixation generated a savings of approximately USD 15.2 billion in the 2019–2020 crop season. In this same season, the soybean cropped area in Brazil using this technology reached around 85% of the total [30].

Another example is the successful integrated management of the *Helicoverpa armigera* caterpillar using biological control, which was adopted when chemical methods proved ineffective and led to significant damage to crops in 2012 in Brazil [2].

In order to analyze how the topic has been dealt with by the scientific community, a bibliometric analysis of co-occurrences was carried out based on a bibliographic survey. To identify publications in the topic “bioinput production in Brazil” in the last years, a bibliographic survey was conducted in the Web of Science, the Scientific Electronic Library Online (SciELO), and Google Scholar databases, covering scientific publications derived from studies conducted. To guide the literature review, the analysis focused on the risks and opportunities associated with bioinputs production in Brazil, especially the on-farm system.

The search strategy included the following terms: “Brasil,” “Brazil,” “bioinsumos,” “bioinputs,” “bio-inputs,” “biopesticides,” “biofertilizers,” “on farm,” “on-farm production,” “own production,” “production for own use,” “biofábricas,” and “biofactories,” combined with the descriptors “agriculture,” “risks,” “human health risk,” “biosafety,” “contaminants,” and “opportunities.” The inclusion criteria were: (1) studies conducted between 2020 and 2024; (2) scientific article format; (3) articles published in Portuguese, Spanish, French or English; and (4) articles meeting the previous criteria that specifically addressed the risks or opportunities of bioinputs production.

In order to understand the relationship among the terms and concepts present in the 30 articles identified through the bibliographic survey, a bibliometric analysis of co-occurrence and temporal overlay was performed using VOSviewer [31]. The conceptual structure reveals possible associations among terms, predominance of occurrence, and emerging trends.

**Fig. 3:**
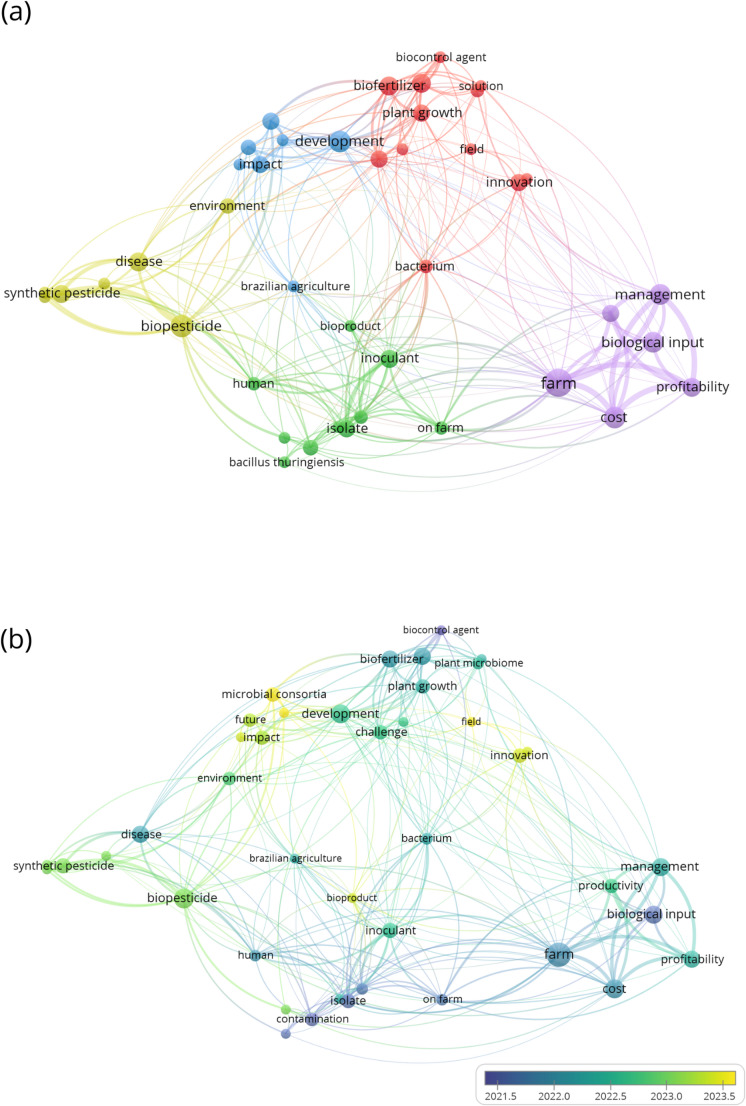
(**a**) Relationship among the most frequently occurring terms in studies related to the production of bioinputs on-farm. (**b**) Visualization of the publications in temporal overlap in the period from 2020 to 2024

Five sets of the most frequently used keywords were found, centered on the terms “biopesticide”, “development”, “plant growth”, “farm” and “inoculant” (Fig. 3a). These were associated with a number of other terms within their respective clusters and also showed numerous connections between the clusters, demonstrating a well-connected network.

When evaluating the time overlap of the publications (Fig. 3b), it can be seen that, in the last years, there have been more publications that perceive bioinputs as innovation, development, and profitability, including the keywords “farm” and “on farm”. Studies relating to concerns about “management” and “contamination” have also become more frequent in recent years. These trends reinforce the central hypothesis of this review that the rapid expansion of bioinputs, particularly under decentralized on-farm production, must be accompanied by an adequate quality control and mechanistic understanding to prevent systemic biosafety failures.

## Brazil’s regulatory framework for bioinputs

**Fig. 4:**
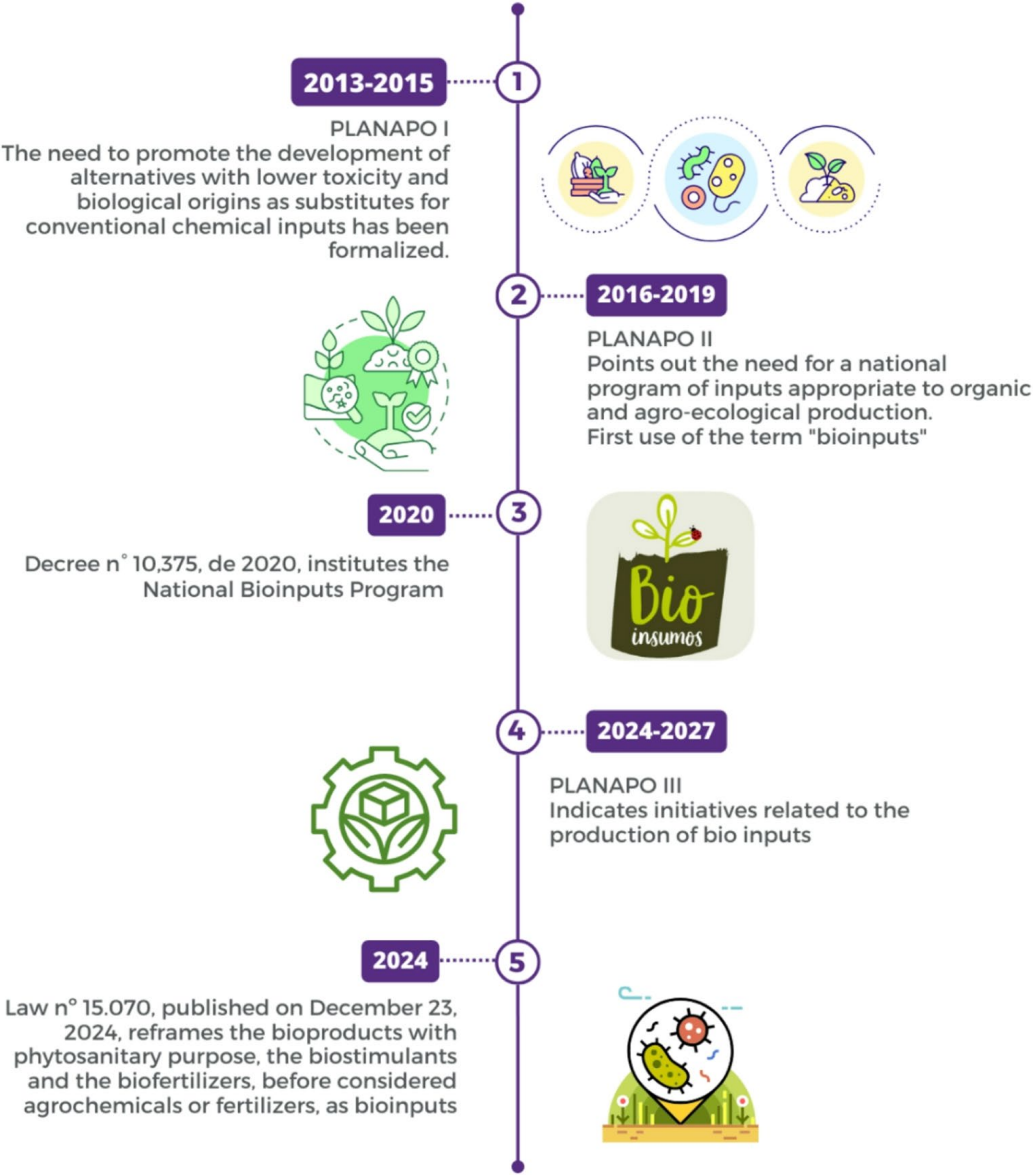
Timeline of the regulatory framework for bioinputs in Brazil

The evolution of bioinputs research and development, adoption by farmers, and commercialization was followed by modification in the regulatory framework (Fig. 4). From a legal perspective, the first definition of bioinputs was established by Decree n^o^ 10,375 of May 26, 2020, which instituted the National Bioinputs Program [32]. Currently, according to the new Bioinputs Law - Law n^o^ 15,070, of December 23, 2024, a bioinput is defined as a product, process or technology of plant, animal, or microbial origin, including that originating from a biotechnological process, or structurally similar and functionally identical to that of natural origin, intended for use in the production, protection, storage and processing of agricultural products or in aquatic production systems or planted forests, which interferes with the growth, development and response mechanism of animals, plants, microorganisms, soil and derived substances and which interacts with physical, chemical and biological products and processes [33].

However, before the regulatory frameworks mentioned above defined bioinput, there was a long process to reach this regulatory breakthrough. Initially, to formalize the encouragement of the development of less toxic alternatives of biological origin to replace conventional chemical inputs, the National Commission for Agroecology and Organic Production (CNAPO), one of the bodies in charge of managing the National Policy and Plan for Agroecology and Organic Production (PLANAPO), included this need in the first version of PLANAPO (2013–2015) [10]. Later on, Goal 6 of Planapo II (2016–2019 cycle) explained the need to create a national program of appropriate inputs for organic and agroecological production (Bioinputs Program), when in fact, the term bioinputs was first used [34] (Fig. 4).

Then, the National Bioinputs Program was created in 2020 by the Decree n^o^ 10,375. The program’s purpose is to expand and strengthen the use of bioinputs in Brazil, and one of its guidelines consists of valuing Brazilian biodiversity, which has great potential to be exploited sustainably [16].

In 2024, the III Planapo was published for the period 2024–2027, established by Interministerial Ordinance MDA/SG-PR/MAPA/MDS/MMA/MS/MCTI n^o^ 7, of October 2, 2024. The current Plan has initiatives related to the production of bioinputs, such as “promoting the qualification of technicians and family farmers for the production and use of bioinputs suitable for organic and agroecological production” and “preparing technical publications aimed at expanding and qualifying the production and use of bioinputs suitable for organic and agroecological production and socio-biodiversity,” which aims to stimulate and encourage the production and use of bioinputs suitable for organic and agroecological production [35]. These initiatives seek to re-establish a focus on the sector from which the initial bioinputs movement originated.

Regarding regulating the production of bioinputs on-farm, there have been two proposals from the Brazilian legislature in recent years. One was the bill (PL) initiated by the Chamber of Deputies, PL 658/21, and the other, PL n^o^ 3,668/21, with the Senate as the initiating House. Both proposed legal provisions to regulate the production of bioinputs for own use within rural properties [36, 37].

One of the obstacles to this regulation for many years was that bioproducts used to control pests and diseases, i.e., for phytosanitary purposes, were still included in the definition of pesticides. Even with the new regulation in force, Law n^o^ 14,785, of December 26, 2023 [38], which repealed the previous Pesticides Law, Law n^o^ 7,802/89, the concept of pesticides still included biological processes:

*“. XXVI - pesticides: products and agents of physical*,* chemical or biological processes intended for use in the production sectors*,* in the storage and processing of agricultural products*,* in pastures or in the protection of planted forests*,* whose purpose is to alter the composition of flora or fauna*,* in order to preserve them from the harmful action of living beings considered harmful;.”* [38] (Author´s Translation from Portuguese).

It wasn’t until the end of 2024 that the bills mentioned above were attached, resulted in the final text sanctioned and became Law n^o^ 15,070, published on December 23, 2024 [33] (Fig. 4). According to this law, bioproducts for phytosanitary purposes or biostimulants are finally considered bioinputs and are no longer pesticides. The law also covers biofertilizers, which are now classified as bioinputs.

Finally, the new regulation establishes that Law n^o^ 14,785, of December 27, 2023, which pertains to pesticides, and Law No. 6,894, of December 16, 1980, which concerns fertilizers, correctives and inoculants, do not apply to bioinputs. However, until this law is regulated, which had a deadline of up to 360 days, the process of new registrations will follow the specific regulations for each type of product. It is noteworthy that, although the aforementioned deadline expired at the end of 2025, the regulation of the new Bioinputs Law has not yet been published.

Thus, the Brazilian legal framework has made significant advancements in recent years, and the country is seen as a benchmark in the regulatory transition of bio-based products. Brazilian legislation can be considered as one of the most advanced in the world [39]. In this context, the regulatory evolution may directly provide conditions whether biofactories will operate as sustainability-promoting infrastructures or as potential sources of biological, environmental and public health risk.

## Advantages of production of bioinputs on-farm

Even before the National Bioinputs Program was established, family farmers have been producing bioinputs on their own farms. More recently, large commodity producers have also started to adopt and manufacture bioinputs for use on their properties, driven by the high demand for this type of product and the notable economic benefits of this practice [40]. However, the commercialization of bioinputs manufactured on the property is prohibited [41].

In addition to the economic advantages, another factor that encourages bioinput on-farm production is the possibility of registration exemption when the product is not for commercial purposes. This regulatory opening began in July 2009, with the publication of Decree n^o^ 6,913 of July 23, 2009, which amended Decree n^o^ 4,074 of 2002 [42]. Paragraph 8 of Article 1 of Decree 6,913 of 2009 stipulated that phytosanitary products approved for use in organic farming and produced exclusively for their own use were exempt from registration. As a result of this regulatory change, there has been a significant increase in the production of bioinputs of microbiological origin on farms [43]. Decree n^o^ 10,833 of October 7, 2021, which amended Decree n^o^ 4,074 of 2002, established that phytosanitary products with approved use for organic farming produced exclusively for their own use in organic or conventional production systems are exempt from registration [44]. This clearly encourages this type of self-production on both organic and conventional farms.

Farmers who implement biofactories on their farms have several advantages, including lower manufacturing, storage, and transportation costs compared with purchasing ready-made commercial products [16, 40]. This cost reduction was demonstrated numerically on a rural soybean-producing property located in Rio Verde, Goiás state [45]. When comparing two harvests with conventional management with two other harvests in which bioinputs manufactured on the farm were applied, it was revealed that production costs were reduced by 58.6%. Other parameters, such as productivity and profitability, grew by 13% and 175%, respectively.

In addition, the on-farm production system represents sovereignty and emancipation for small and large Brazilian producers [46], offering autonomy due to independence from exchange rate fluctuations and the foreign market, as well as non-dependence on commercial bioproducts, which remain very expensive. Furthermore, in some Brazilian regions, commercial bioproducts are difficult to access. It is also worth noting that products based on living organisms often require cold storage to maintain their viability [46–48].

## Challenges in the on-farm production of bioinputs and strategies to them

Given that the on-farm production of bioinputs involves the manipulation of living organisms, this type of activity cannot be immediately considered low-risk. Microorganisms may pose several risks to humans, including pathogenic infections, toxin production, and allergen sensitization. These risks are particularly relevant in the context of plant-beneficial microorganisms used in agriculture, where they can affect workers and end-users. Furthermore, the potential for bacteria to carry antibiotic resistance genes is a significant concern, as it could worsen the issue of antimicrobial resistance. These risks necessitate careful consideration and management in the production and application of such microorganisms [49].

Additionally, it encompasses various technologies, processes, and products, each with its unique characteristics and associated potential risks. Therefore, certain precautions must be taken during the manufacturing of these products to ensure their quality and safety for both human health and the environment [28, 36]. Various studies have raised concerns about on-farm production, highlighting issues related to producer safety, environmental safety, and risks to the population’s health. Table 2 summarizes potential dangers of on-farm production.

**Table 2:** Risks associated with the production of bioinputs on-farm

Main risks	Conter measure	Reference
Occupational diseases related to conidia in production systems based on entomopathogenic fungi. Possible proliferation of bacteria and fungi pathogenic to humans depending on the type of fermentation.	Personal protective equipment	[27]
The target bioinpout microorganism may produce metabolites toxic to humans. Raw vegetables in which *Bacillus thuringiensis-*based bioinput was applied presented toxins harmful to humans.	Strict control of the toxins produced during the production process.	[50]
Contamination with pathogenic bacteria, especially of the genera *Enterobacter*,* Citrobacter*,* Klebsiella*, and *Escherichia*, may lead to contaminated food products, especially concern for leafy vegetables.	Control during the production process.	[51]
Ineffectiveness of manufactured bioinputs, which may increase the number of non-target microorganisms, and reduce the prevalence and viability of the one of interest.	Trained personnel to identify contaminants and unwanted organisms.	[59]
Signaling the acquisition of antibiotic-resistance genes.	Trained personnel to identify contaminants and unwanted organisms.	[59]

The producer’s safety needs to be carefully considered when producing a bioinput. As each microorganism possesses unique characteristics that can be harmful to human health, the potential occupational diseases that may affect employees in a biofactory should be considered before its multiplication in biofactories. For instance, personnel can be exposed to conidia dispersed in the air when handling entomopathogenic fungi [27]. For this reason, personal protective equipment must be properly used. Additionally, if on-farm production is not appropriately conducted, fungi of the *Aspergillus* and *Fusarium* genera may proliferate in solid-state fermentations. In liquid multiplication in an open system, concerns arise about pathogenic bacteria to humans and/or toxic metabolites produced by them [27].

Concern also arises when considering the food the population will consume *in natura*. Thus, bioinputs selection, multiplication and usage (including target crop) should be carefully controlled to avoid foodborne outbreaks (FBO). The use of insecticide products based on *Bacillus thuringiensis* (Bt) in vegetables was associated with cases of FBO in France [50]. Out of 250 cases investigated, 49 were found to have Bt contamination in vegetables. In 19 of these 49 FBO, Bt was the unique microorganism detected, indicating it as the probable causative agent. The research showed that more than 50% of the Bt isolates were detected in raw vegetables, mainly tomatoes (48%), which was the source of contamination in these cases. Considering the pathogenic potential of the bacteria, it is necessary to strictly control the toxins produced during the production process. If a Bt strain that produces β-exotoxin spreads, there are risks to human health since this toxin is highly toxic to mammals [50].

Similarly, a bionput formulation based on a pool of microorganisms obtained from the environment, produced for an organic farming experimental area located in Brazil, demonstrated contamination with bacteria of the Enterobacteriaceae family [51]. Bacteria from the genera *Enterobacter*,* Citrobacter*,* Klebsiella*, and *Escherichia* were the most commonly identified in the analyzed formulation at all time samples. The presence of these microorganisms in the formulation may indicate a pathogenic potential, as several species in this group are associated with human diseases. This becomes even more worrying if they are used in the production of leafy vegetables that are eaten raw [51].

In fact, the literature shows that in open or semi-open fermentation systems, fast-growing facultative anaerobes, such as Enterobacteriaceae, often outcompete the target microorganisms due to their metabolic versatility and rapid substrate uptake, leading to dominance shifts in the microbial consortium. This competitive displacement directly compromises bioinput efficacy and increases biosafety risks [52, 53]. These bacteria also display robust biofilm-forming capacity on fermentation vessel surfaces, which facilitates persistent contamination cycles and cross-batch carryover. Such biofilm-mediated persistence can explain the recurrent detection of these in contamination cases and represents a mechanistic bottleneck for maintaining microbiological purity [54, 55].

Still on the possible risks of bioinputs applied to crops that produce food to be consumed *in natura*, microalgae-based bioinputs should be highlighted. Despite the potential for using microalgae species like *Asterarcys quadricellulare*,* Chlorella vulgaris* and *Scenedesmus obliquus* as bioinputs in agriculture [56, 57], the toxins produced by some species are known to be important in terms of public health. Oliveira & Bragotto [58] warn about the conditions under which microalgae-based products are produced. The material obtained from microalgae and its potential dangers is closely associated with the specific characteristics of these microorganisms or the components present during cultivation, including the stages of development and the conditions under which the biomass is produced. Thus, understanding the potential health impacts of using microalgae-based products requires proper identification of the species involved, as well as monitoring for any toxins and allergenic substances that may be present.

The ineffectiveness of bioinputs can occur due to several reasons; however, in on-farm production, the risk of contamination and loss of viability of the target microorganism should be particularly considered. In a recent study, 18 samples from bioinputs produced on-farm located in five Brazilian states were collected and analyzed for contaminants. All the samples were contaminated with non-target microorganisms and the target microorganisms were not detected in most of the samples [59]. The product shall not demonstrate the expected activity, and the efficacy of bioproducts and the target microorganism may be questioned due to operational errors in the production process. An example of such a problem occurred with the Brazilian program for the use of *Baculovirus anticarsia* to control the soybean caterpillar *Anticarsia gemmatalis* [60]. As the baculovirus is pest-specific and has been proven effective, farmers have used it on millions of hectares of soybeans to control the caterpillar, avoiding the use of synthetic insecticides.

Faced with this success, farmers started to collect infected caterpillars and store them to be used in the next crop season. However, the lack of quality control in terms of purity and concentration of *Baculovirus anticarsia* resulted in bad control of the pest, so the strategy ultimately ended up being discredited and contributed to the early termination of this program [60].

Thus, on-farm production of bioinputs must adhere to minimum procedures during the cultivation of the target microorganism to ensure that the organism of interest prevails in the culture medium. Inadequate control of temperature, oxygen availability, pH and accumulation of inhibitory metabolites can reduce target microorganism viability and favor contaminant proliferation, directly linking process design to product safety and field efficacy [61–63].

Another concern critical in on-farm production, where microbial contamination is more susceptible than in industrial facilities, is the acquisition of antibiotic-resistance genes by bacteria. In the same study [59], 61% of the samples showed contamination by bacteria of the *Enterococcus* genus. These bacteria have significant public health importance and are associated with bacteremia, septicemia, infections, abscesses, meningitis, and endocarditis. Through molecular analysis of 85 isolates, they found that many of the isolated organisms (almost 30%) were carrying antibiotic resistance genes. Among 34 isolates representing genera that are potentially pathogenic to humans, six showed intrinsic resistance, and 18 of them showed acquired resistance to at least one type of antibiotic. Finally, it has been pointed out that pathogenic species, in addition to having multiple resistance to antimicrobials, can promote the dispersal of resistance genes into the environment [59].

The presence of microorganisms with antibiotic resistance in agricultural production systems and throughout the food life cycle poses a serious threat to human and animal health [64]. Studies show that horizontal gene transfer mediated by plasmids, transposons and integrons can facilitate the acquisition and dissemination of antimicrobial resistance genes among co-cultivated bacteria in fermentative processes, particularly under selective pressures imposed by high cell densities, prolonged co-cultivation periods and nutrient-rich fermentation environments [65–67]. Then, these processes may facilitate the spread of antimicrobial resistance determinants and transform biofactories into potential amplification points for resistance genes within agroecosystems.

**Fig. 5:**
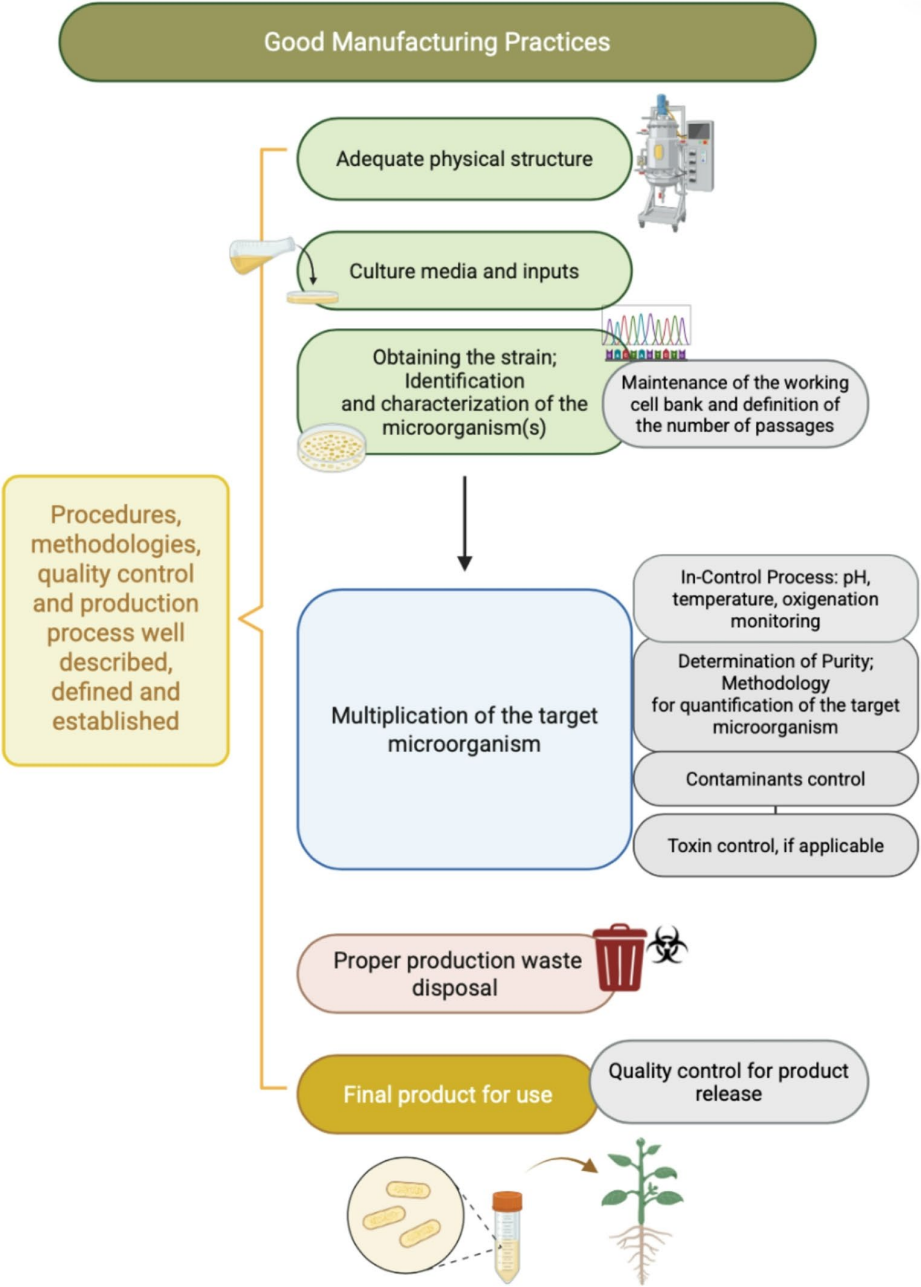
Criteria and parameters for implementing good manufacturing practices in biofactories producing bioinputs

To ensure the supply of safe food to the population and maintain the credibility on bioinputs technology, which has been developed over many decades, it is clear that on-farm producers need to implement minimum steps to control process quality. The precise identification of the microorganism used in the production of the bioinput, the monitoring of possible contaminants, especially those pathogens to plants, humans and animals, and the quantification of the target organism to ensure the defined concentration to guarantee the efficiency of the bioinput, are the minimum. Considering the general principles of good manufacturing practices and the critical control points associated with bioinput production previously discussed, the minimum parameters to observe in biofactories are summarized in Fig. 5.

Also, it is important to note that key scientific gaps remain regarding microbial community dynamics in non-sterile biofactories, long-term stability of target strains across production cycles, and environmental persistence of non-target microorganisms.

## Prospects and challenges for on-farm production of bioinputs in Brazil

Brazil’s vast biodiversity provides a powerful biotechnological arsenal and serves as a strategic tool for agribusiness [46]. For decades, significant efforts have been made to enhance the use of this biodiversity for sustainable agriculture development, which is now evident in the vast array of available products, the various technological packages developed, and large-scale production and application of bioinputs. This situation positions Brazil as an international leader in bioinputs [16].

The global financial outlook for bioinputs is positive and optimistic, as the sector is expected to experience growth. In 2022 the biopesticide market had a turnover of 6.51 billion dollars; in the same year, the bio-fertilizer market had a turnover of 2.2 billion dollars, and the biostimulant market, 3.14 billion dollars, all with estimates of a growth rate of more than 10% by the year 2029 [7]. Investment in research and development in this area is necessary and growing, with an increasing number of publications and registered products (Fig. 2). Public research institutions, such as the Brazilian Agricultural Research Corporation (Embrapa) and Universities in Brazil, should continue to play a crucial role in generating technical and scientific knowledge regarding the discovery of new active ingredients and formulations of bioinputs [7, 46].

Further innovations in the bioinputs sector are expected to result from investments in product development. Products on a nanotechnology platform, phytovaccines based on recombinant proteins, pesticides based on plant extracts, products based on interfering RNA, biofertilizers with a pool of organisms, and products based on microorganisms’ metabolites are among the promising ones [7, 22].

The great success in the development and the adoption of bioinputs in Brazilian agriculture has driven the development of products based on new microorganisms, the increasing on product registrations (Fig. 2), and the establishment of biofactories. However, rapid biotechnological development has also presented challenges in different fields, such as the public sector’s difficulty in proposing regulations that account for current advances.

As mentioned previously, the regulations have evolved significantly in recent years (Fig. 4), but critical regulations regarding biofactories are still pending release. To achieve a solid market expansion, there is a need to regulate and manage on-farm production, ensuring the quality and safety of the bioinputs used and guaranteeing that the associated technology is not discredited [10]. In this context, a challenge is to develop regulations that take into account the different structures available to both small and large producers who manufacture inputs for their own use. The participation of civil society and other relevant stakeholders in discussions about regulations for bioinputs is crucial, as their absence can be detrimental to the creation and implementation of effective public policies [36].

In this context, raising awareness among farmers who produce bioinputs on-farm about the risks associated with such activity (Table 2) may further positively impact the development of biofactories. The importance of technical and operational support, and knowledge about biological safety is emphasized, with education, training, and behavioral change as tools to prevent biological accidents [68, 69].

One of the structuring actions of the Brazilian National Bioinputs Program is to encourage the development of manuals on good practices for the production, use and application of bioinputs in partnerships with public and private institutions [70]. Decree n^o^ 10,375/2020 determined the publication of a manual of good practices aimed at bioinput production units, to be promoted throughout the country. However, this manual has not been published so far. If published, this document would represent an important tool to guide rural producers [28]. The absence of enforceable of Good Manufacturing Practices (GMP) manuals and harmonized national monitoring protocols currently represents a structural vulnerability of the Brazilian on-farm bioinput sector.

Even though the GMP manual mentioned above has not yet been made available, it is worth mentioning that there are some publications developed by Embrapa [71–73] and by the regulated sector itself [74] with recommendations on how to conduct quality control of bioinputs.

Regarding good practices for bioinputs production, the new Law n^o^ 15,070 of 2024 states that the manufacturing of bioinputs in a production unit for own use must adhere to the guidelines established by the Federal Agricultural Defense Agency. Another provision of this law ensures the ongoing production of bioinputs for own use, as well as the supply of necessary inputs, until the regulations and guidelines for good practices are enacted. Once the good practice guidelines are published, producers must comply within twelve months [22]. In other words, despite the new law, many producers still lack proper guidance on good manufacturing practices.

Despite this gap, it’s important to note that Law n^o^ 15,070 of 2024 introduced a significant advance regarding the strains of microorganisms to be used in on-farm multiplication. According to this law, rural producers who produce bioinputs for their own use containing a microorganism as the active ingredient are exempt from registering isolates or strains in the National System for the Management of Genetic Heritage and Associated Traditional Knowledge as long as these are acquired from public or private germplasm banks or from registered bioinput inoculums [33]. Thus, the bioinputs manufactured by these producers can be made from microorganisms obtained directly from germplasm banks accredited by the Federal Agricultural Defense Agency, from registered inoculums, or from communities of organisms collected on-site, in the latter case, production must occur exclusively by multiplication and application on site, with specific criteria for transportation between properties defined by the competent agency [33].

Another challenge and opportunity, which can be minimized by public policies, is the need to encourage the establishment of bioinput production units in the North and Northeast regions of the country. Considering the survey of on-farm biofactories in Brazil carried out by Xavier and Rodrigues [29], there was a gap in the occurrence of biofactories in these regions, which in turn demands attention due to the vulnerable food security scenario in some of their states. The expansion of biofactories in those regions may favor economic development through sustainable production.

In addition to regulatory topics, future public policies and research programs should also evolve to reduce the risks, increase sustainability and close scientific gaps regarding bioinputs production, use and impact on the human health and on the environment. Nationwide surveillance initiatives and standardized microbiological testing of commercial and on-farm products should be prioritized. Easy and fast to use molecular tests to assure target species propagation and identification of possible contaminants would increase product safety and efficiency. Finally, mechanistic studies on microbial interactions, large-scale monitoring of bioinputs and life-cycle assessments should be carried out to sustain sustainability, credibility and positive impact on the environment.

## Conclusions

The implementation and increased use of bioinputs in agriculture require proper management to maximize the benefits of these products while minimizing the associated risks. The production of bioinputs adhering to good manufacturing practices is essential to ensure the quality and safety of these inputs. To this end, on-farm production, more specifically, requires assertive regulation to cover both small and large producers, as well as guidance and parameterization of production processes.

Based on the above, responsible bodies and producers must be able to identify the root cause of potential health, environmental, and occupational problems, thereby enhancing the credibility of the production sector and ensuring safe food for the population. In this way, Brazil can continue to lead the global arena with successful results, considering sustainability, productivity, and socio-economic aspects.
